# Duck plague virus gE serves essential functions during the virion final envelopment through influence capsids budding into the cytoplasmic vesicles

**DOI:** 10.1038/s41598-020-62604-9

**Published:** 2020-03-27

**Authors:** Tian Liu, Mingshu Wang, Anchun Cheng, Renyong Jia, Qiao Yang, Ying Wu, Mafeng Liu, Xinxin Zhao, Shun Chen, Shaqiu Zhang, Dekang Zhu, Bin Tian, Mujeeb Ur Rehman, Yunya Liu, Yanling Yu, Ling Zhang, Leichang Pan, Xiaoyue Chen

**Affiliations:** 1 Institute of Preventive Veterinary Medicine, Sichuan Agricultural University, Wenjiang, 611130 People’s Republic of China; 2 Avian Disease Research Center, College of Veterinary Medicine of Sichuan Agricultural University, Wenjiang, 611130 People’s Republic of China; 3 Key Laboratory of Animal Disease and Human Health of Sichuan Province, Sichuan Agricultural University, Wenjiang, 611130 People’s Republic of China

**Keywords:** Herpes virus, Mutation

## Abstract

Duck plague virus (DPV), a member of the *alphaherpesviruses* subfamily, causes massive ducks death and results in a devastating hit to duck industries in China. It is of great significance for us to analyze the functions of DPV genes for controlling the outbreak of duck plague. Thus, glycoproteins E (gE) of DPV, which requires viral cell-to-cell spreading and the final envelopment in herpes simplex virus 1 (HSV-1) and pseudorabies virus (PRV), was chosen herein. The *gE* mutant virus BAC-CHv-ΔgE was constructed by using a markerless two-step Red recombination system implemented on the DPV genome cloned into a bacterial artificial chromosome (BAC). Viral plaques on duck embryo fibroblast (DEF) cells of BAC-CHv-ΔgE were on average approximately 60% smaller than those produced by BAC-CHv virus. Viral replication kinetics showed that BAC-CHv-ΔgE grew to lower titers than BAC-CHv virus did in DEF cells. Electron microscopy confirmed that deleting of DPV *gE* resulted in a large number of capsids accumulating around vesicles and very few of them could bud into vesicles. The drastic inhibition of virion formation in the absence of the DPV *gE* indicated that it played an important role in virion morphogenesis before the final envelopment of intracytoplasmic nucleocapsids.

## Introduction

Duck plague virus (DPV), the causative agent of duck plague disease in its natural hosts ducks, geese, and swans, has been classified as a member of the subfamily *Alphaherpesvirinae* of the family *Herpesviridae*, which includes herpes simplex virus (HSV) types 1 and 2 (HSV-1 and HSV-2), pseudorabies virus (PRV), varicella-zoster virus (VZV), and Marek’s disease virus (MDV)^1^. DPV can cause focal necrosis in the parenchymal organs of ducks and increase vascular permeability, which results in massive petechial hemorrhaging in the parenchymal organs, lymphoid, and digestive tract and causes large numbers of duck deaths^2^.

The morphology of DPV virions purified by sucrose density gradient centrifugation has been observed by electron microscopy, revealing that purified DPV virions are round in shape with a diameter of approximately 150 ~ 200 nm. Under high magnification, four morphological structures, including the core, capsid, tegument, and envelope, are observed in DPV virions^3^. Similar to other alphaherpesviruses, DPV has a linear double-stranded DNA genome and an icosahedral capsid shell comprising the inner nucleoprotein core, which is surrounded by a tegument layer and a lipid envelope. However, research on the DPV assembly process is progressing more slowly than research on other alphaherpesviruses. The capsid formation process in the nuclei of DPV-infected cells and the mechanisms of tegumentation and envelopment have not been systemically or comprehensively investigated. Although DPV *gE* gene has been identified, few studies explored its functions^4^.

Glycoproteins E (gE) is not essential for the extracellular entry of HSV and PRV but plays a pivotal role in directing virion spreading from cell-to-cell in polarized cells, such as epithelial cells and neuronal cells^5–8^. The mutant lacking *gE* displayed a small plaque phenotype, and most of the enveloped virions accumulated on the cell apical surface rather than on the lateral surface, in contrast to the wild-type virus^9^. The gE cytoplasmic (CT) domain could facilitate cell-to-cell spreading by trafficking virions through the trans-Golgi network (TGN) to the lateral surfaces of polarized cells, and the extracellular (ET) domains of gE could bind ligands at cell junctions to promote virus entry into adjacent cells^9–11^. Furthermore, gE plays essential roles in the replication of specific alphaherpesviruses, such as MDV-1 and VZV^12–14^. gE also provides essential but redundant functions during the acquisition of the final virion envelope. In HSV-1, gE together with gD affects the production of enveloped virus particles^15^. Similarly, in PRV, extracellular and intracytoplasmic enveloped virus particles are absent in the absence of *gE* and *gM* genes, and unenveloped capsids accumulate in the cytoplasm surrounded by tegument proteins in infected cells^16^. However, the single deletion of *gE* only slightly reduces the number of enveloped virions in both HSV- and PRV-infected cells^15,16^.

To explore the functions of DPV gE in-depth, *gE* gene was deleted by using a markerless two-step Red recombination system implemented on the DPV genome that was cloned into a bacterial artificial chromosome (BAC)^17^. The viral plaques on the duck embryo fibroblast (DEF) cells of the *gE* mutant virus were smaller than those produced by the BAC-CHv virus. Intracellular and extracellular viral replication analyses showed that the *gE* mutant grew to lower titers than the BAC-CHv virus in DEF cells. Electron microscopy analysis confirmed that deleting DPV *gE* impaired the final viral envelopment, during which fewer capsids could bud into cytoplasmic vesicles. These results indicated that DPV gE played a significant role in the final envelopment process and provided new information for the study of the molecular mechanism of DPV.

## Results

### Construction and characterization of the recombinant virus BAC-CHv-ΔgE

**Figure 1:**
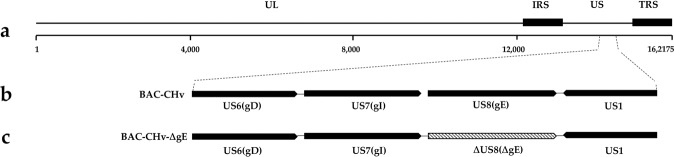
Structure of the DPV genome and construction of *gE* mutant virus. (**a**) Diagram of the DPV genome. It is divided into the unique long (UL) and unique short (US) regions flanked by the terminal repeat (TR) and internal repeat (IR) regions. (**b**) Shows expanded genomic regions of the US6 (gD), US7 (gI), US8 (gE) and US1 open reading frames. (**c**) Shows the deletion of the whole *gE* open reading frame.

To characterize the functions of DPV gE, we successfully constructed a mutant derived from a BAC copy containing the CHv strain genome in which the *gE* coding sequence was deleted by a markerless two-step Red recombination system; the mutant was named BAC-CHv-ΔgE **(**Fig. 1a–c**)**. To confirm that nonspecific mutations occurred at the rest part of DPV genome, the revertant virus was constructed, namely BAC-CHv-ΔgE Rev, as described in the Materials and Methods. PCR products containing regions upstream and downstream of the *gE* coding sequence were produced and sequenced (data not shown). The correctly sequenced plasmids were transfected into DEF cells, and green fluorescence was observed.

**Figure 2:**
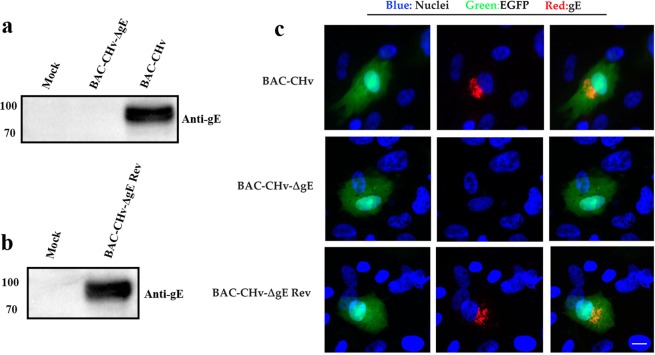
Identification of the expression of gE. (**a**) Western blot analysis of the gE expression of BAC-CHv-∆gE. (**b**) Western blot analysis of the gE expression of BAC-CHv-∆gE Rev. (**c**) Indirect immunofluorescence analysis of gE expression.

To assess the expression of gE, DEF cells were infected with BAC-CHv and its mutant and revertant viruses. Cells were lysed with RIPA buffer, and the extracts were subjected to Western blot analysis. According to the Western blot results, BAC-CHv and BAC-CHv Rev expressed gE at approximately 100 kDa and BAC-CHv-ΔgE failed to express gE **(**Fig. 2a,b). The indirect immunofluorescence assay (IFA) revealed no gE expression in DEF cells when infected with BAC-CHv-ΔgE **(**Fig. 2c**)**. Notably, the subcellular localization of gE protein presented a punctate distribution with a marked juxtanuclear localization in DEF cells **(**Fig. 2c**)**.

### gE protein is implicated in viral cell-to-cell spreading

**Figure 3:**
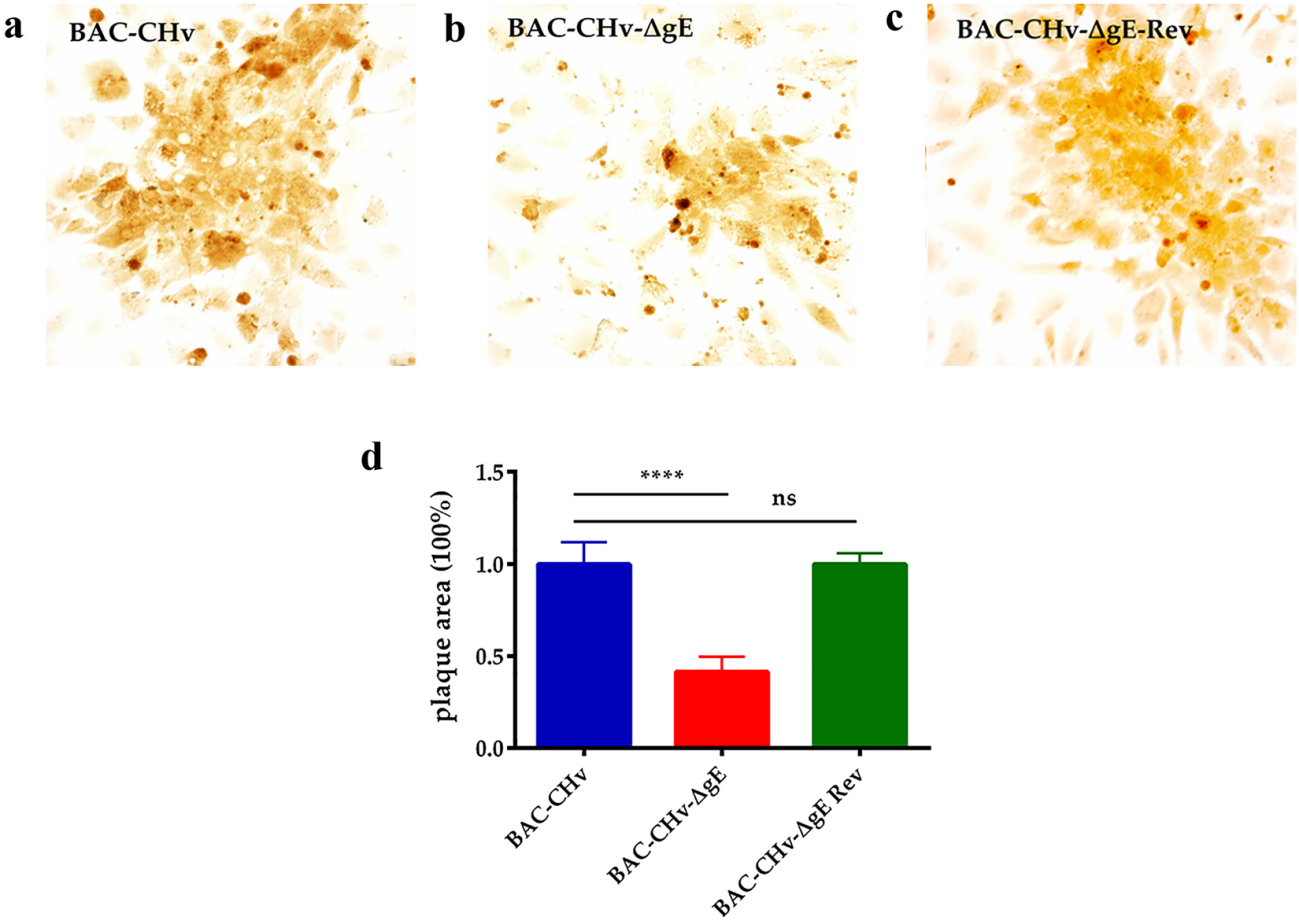
Plaque morphologies of BAC-CHv, BAC-CHv-∆gE and BAC-CHv-∆gE Rev. DEFs were infected with each virus, and viral plaques were visualized by immunohistochemistry using the polyclonal rabbit anti-CHv antibody at 24 h post-infection. (**a**) Plaque morphologies of BAC-CHv-infected cells. (**b**) Plaque morphologies of BAC-CHv-∆gE-infected cells. (**c**) Plaque morphologies of BAC-CHv-∆gE Rev-infected cells. (**d**) Statistical analyzation of plaque morphologies of BAC-CHv, BAC-CHv-∆gE and BAC-CHv-∆gE Rev. Error bars represent the standard errors of the means.

Glycoproteins of alphaherpesvirus play an important role in viral cell-to-cell spreading. We performed plaque morphology assays in this paper to explore whether the *gE*-deleted virus had effects on the transmission of viruses between adjacent cells. The plaque morphologies of BAC-CHv, BAC-CHv-∆gE and BAC-CHv-∆gE Rev mentioned above were examined on DEF cells at 24 h post-infection. As expected, the BAC-CHv produced the largest plaques, while BAC-CHv-ΔgE produced smaller plaques. The deletion of *gE* caused the production of viral plaques that contained an average value of 41.4% compared with plaques formed by BAC-CHv, which was significantly different (p ≤ 0.0001) **(**Fig. 3a,b,d**)**. The revertant formed plaques that were similar in size to those of BAC-CHv, and the differences were not significant **(**Fig. 3c,d). These results demonstrated that DPV gE influenced viral cell-cell spreading.

### Deleting *gE* inhibits viral growth at the late time of infection

**Figure 4:**
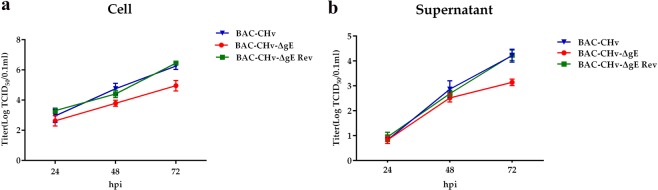
Viral growth curves of BAC-CHv, BAC-CHv-∆gE and BAC-CHv-∆gE Rev. DEFs were infected with BAC-CHv, BAC-CHv-∆gE or BAC-CHv-∆gE Rev at an MOI of 0.01. The viral titers of infected cells and supernatants at 24 h, 48 h and 72 h post-infection were determined. (**a**) Cell titers of BAC-CHv, BAC-CHv-∆gE and BAC-CHv-∆gE Rev-infected cells. (**b**) Supernatant titers of BAC-CHv, BAC-CHv-∆gE and BAC-CHv-∆gE Rev-infected cells. The data are presented as the mean ± standard deviation (SD) of three independent experiments.

For further exploring the functions of gE, the viral growth curve was performed. DEF cells were infected with BAC-CHv, BAC-CHv-∆gE and BAC-CHv-∆gE Rev, and the viral growth curve of infected cells and their supernatant were performed respectively as described in Materials and Methods. At 24 h postinfection, the viral titers of the *gE*-deleted virus were similar to those of the wild-type virus in cells and supernatants **(**Fig. 4a,b). At 48 h post-infection, the viral titers of BAC-CHv-∆gE were similar to those of BAC-CHv in supernatants but lower than those of the wild-type virus in cells **(**Fig. 4a,b). At 72 h post-infection, the production of viral titers of *gE*-deleted virus was less efficiently with nearly 1 log lower than those of BAC-CHv in cells and supernatants **(**Fig. 4a,b). The growth curve of the revertant virus was the same as BAC-CHv, indicating that deleting *gE* had no effects on the rest of the viral genome. From those results, we concluded that deleting *gE* inhibited viral growth at the late time postinfection.

### Deleting *gE* causes a large number of nucleocapsids accumulated around intracellular vesicles with very few of them budding into vesicles

**Figure 5:**
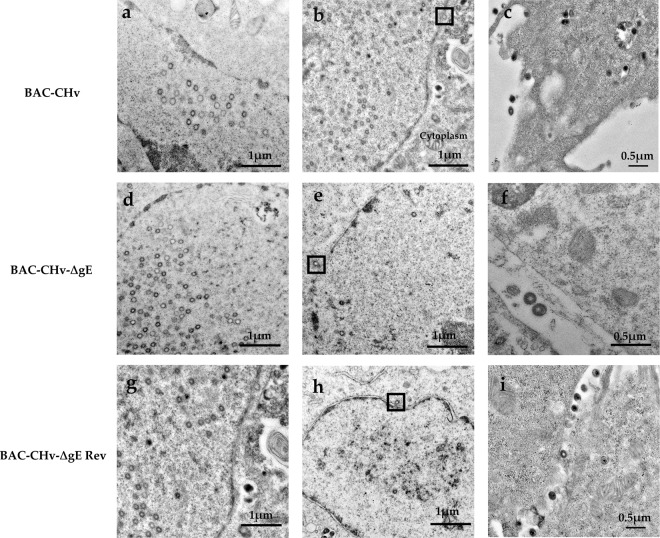
Ultrastructural morphologies of BAC-CHv, BAC-CHv-∆gE and BAC-CHv-∆gE Rev. DEF cells infected with BAC-CHv, BAC-CHv-∆gE and BAC-CHv-∆gE Rev for electron microscopy at 20 h post-infection were shown. (**a**) Nucleocapsids accumulated at nuclear in BAC-CHv-infected cells. (**b**) Primary envelopment process in BAC-CHv infected cells and the process of nucleocapsids de-envelopment at outer nuclear membrane was marked by a black box. (**c**) BAC-CHv viruses released to extracellular space. (**d**) Nucleocapsids accumulated at nuclear in BAC-CHv-∆gE infected cells. (**e**) Primary envelopment process in BAC-CHv-∆gE-infected cells and the process of nucleocapsids de-envelopment at outer nuclear membrane was marked by a black box. (**f**) BAC-CHv-∆gE viruses released to extracellular space. (**g**) Nucleocapsids accumulated at nuclear in BAC-CHv-∆gE Rev infected cells. (**h**) Primary envelopment process in BAC-CHv-∆gE Rev infected cells and the process of nucleocapsids de-envelopment at outer nuclear membrane was marked by a black box. (**i**) BAC-CHv-∆gE Rev viruses released to extracellular space.

**Figure 6:**
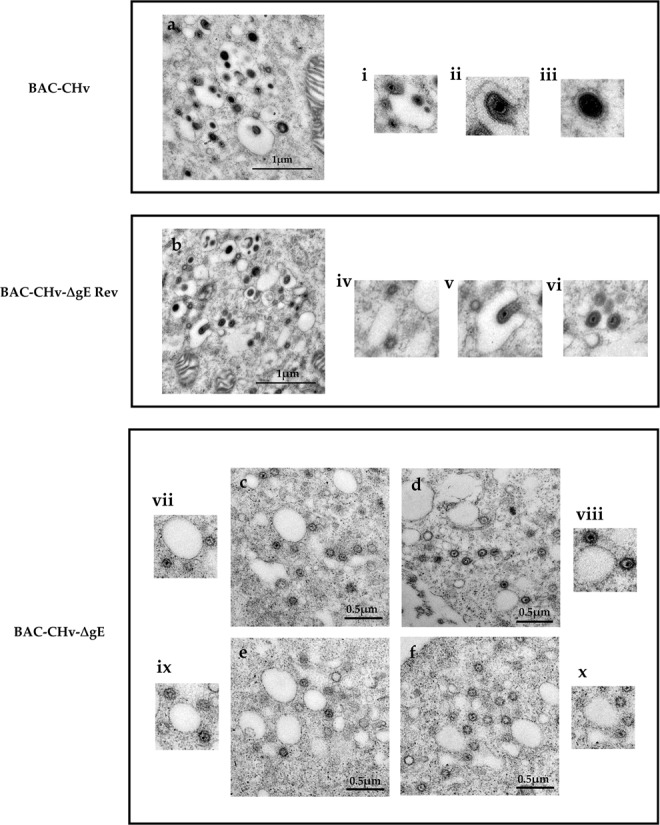
Ultrastructural morphologies of BAC-CHv, BAC-CHv-∆gE and BAC-CHv-∆gE Rev. Electron micrographs of DEF cells infected with BAC-CHv, BAC-CHv-∆gE and BAC-CHv-∆gE Rev for electron microscopy at 20 h post-infection were shown. (**a**) The final envelopment of BAC-CHv. i: nucleocapsids anchored onto the cytoplasm vesicle; ii: nucleocapsids budding into the cytoplasm vesicle; iii: complete virions in the cytoplasm. (**b**) The final envelopment of BAC-CHv-∆gE Rev. iv: nucleocapsids anchored onto the cytoplasm vesicle; v: nucleocapsids budding into the cytoplasm vesicle; vi: complete virions in the cytoplasm. (**c**–**f**) The final envelopment of BAC-CHv-∆gE. vii-x: nucleocapsids anchored onto the cytoplasm vesicle.

Electron microscopy was used to explore the differences in the viral growth of BAC-CHv, BAC-CHv-∆gE and BAC-CHv-∆gE Rev. Pivotal points, including viral particle primary envelopment, de-envelopment, and the final envelopment as well as mature viral particle release, were evaluated **(**Figs. 5 and 6**)**. To this end, DEF cells were infected with BAC-CHv, BAC-CHv-∆gE and BAC-CHv-∆gE Rev at an MOI of 5. Twenty hours after infection, the cells were fixed, stained, and analyzed.

As expected, a complete assembly and egress process was exhibited in BAC-CHv and BAC-CHv-∆gE Rev-infected DEF cells **(**Figs. 5a–c,g–i and 6a,b**)**. Simply, the formation of capsids and loss of primary envelope from the outer leaflet of the nuclear membrane were characterized **(**Fig. 5a,b,g and h**)**. Subsequently, the final envelopment was occurred in the cytoplasm **(**Fig. 6a,b**)**. The details of the final envelopment of BAC-CHv and BAC-CHv-∆gE Rev including nucleocapsids anchored onto the cytoplasm vesicle, nucleocapsids budding into the cytoplasm vesicle, as well as envelopment virions surrounding by the vesicle membrane were also detected **(**Fig. 6a i–iii,b iv–vi**)**. Finally, we also found mature viral particles released into extracellular space **(**Fig. 5c,i**)**.

Ultrastructural analysis revealed that in the absence of *gE*, the process of nucleocapsids formation in nuclear and the egress process of nucleocapsids in the nucleus compartment were similar to BAC-CHv **(**Fig. 5d,e**)**. The number of nucleocapsids accumulated in the nuclear of BAC-CHv-∆gE was similar to BAC-CHv **(**Table 1**)**. However, the final envelopment process of BAC-CHv-∆gE was different from BAC-CHv. In the cytoplasm, numerous aggregated capsid particles were observed surrounding cytoplasmic vesicles, and few of them budded into vesicles in BAC-CHv-∆gE-infected cells and for exploring this issue much more comprehensively, we showed different cells that could descript the same phenomenon **(**Fig. 6c–f**)**. The complete final envelopment process was difficult to detect in BAC-CHv-∆gE-infected cells. To further explore this phenomenon, the distribution of enveloped and nonenveloped virion structures in the cytoplasm was qualified by counting viral particles in cells infected with either BAC-CHv and BAC-CHv-ΔgE, and the average numbers of different structures in the infected cells were listed in Table 1. Approximately 10-fold fewer enveloped particles were found in the cytoplasm of cells infected by BAC-CHv-ΔgE compared with the number in BAC-CHv-infected cells. Moreover, approximately 10-fold fewer enveloped particles were found on the surfaces of cells infected by BAC-CHv-ΔgE than on BAC-CHv-infected cell surfaces **(**Table 1**)**. From the results, we could conclude that DPV gE was implicated in the viral final envelopment process and this may be the main reason for the decrease in the titers of BAC-CHv-ΔgE.

**Table 1:** Distribution of virus particles produced by BAC-CHv and the gE mutant and revertant viruses^*a*^.

Virus	Nucleus	Cytoplasm		Cell surface
	Nonenveloped	Nonenveloped	Enveloped	Enveloped
BAC-CHv	36 (426)^b^	10 (168)	75 (901)	22 (242)
BAC-CHv-**∆**gE	30 (331)	39 (394)	6 (92)	3 (31)
BAC-CHv-**∆**gE Rev	32 (416)	12 (204)	73 (950)	20 (204)

^a^DEF cells were infected with BAC-CHv and the *gE* mutant and revertant viruses for 20 h. The cells were fixed, sectioned, and then examined by electron microscopy.

^b^The numbers of unenveloped nucleocapsids or enveloped virions were counted in approximately 10 to 20 randomly chosen cells. The numbers in parentheses are the total numbers of particles.

## Discussion

During alphaherpesvirus infection, several important processes including DNA replication, capsids formation, viral genome DNA packaging into the capsids, nucleocapsids primary envelopment, nucleocapsids de-envelopment and tegument capsids final envelopment, as well as mature virions release were established^18^. Important virus-encoded (glyco)proteins participate in those processes^18,19^. Nonessential glycoproteins and tegument proteins have been proven to play important roles in viral final envelopment^15,20,21^. DPV, a member of the alphaherpesviruses subfamily, also paths through the same virion assembly process during its infection in DEF cells **(**Figs. 5 and 6**)**. However, only glycoprotein J (gJ) of DPV was confirmed by now that plays an important role in the virion assembly process. DPV deleting gJ produced and accumulated high levels of anuclear particles in the nuclear and cytoplasm which slightly impaired in viral replication^22^. The effects of other DPV proteins on viral assembly processes have not been studied. To further explore virion assembly processes of DPV, in present study, a nonessential glycoprotein gE was identified and functionally characterized by using a specific viral deletion mutant.

DPV gE is a transmembrane protein that has been proved to cluster within a single monophyletic clade through the construction of a phylogenetic tree including fifteen gE protein reference sequences of alphaherpesviruses^23^. DPV *gE* gene is expressed most abundantly during the late phase of the replication cycle. The molecular masses of gE appeared more than 40 kDa higher than the values calculated from the deduced amino acid sequences, a difference which might be caused by the glycosylation **(**Fig. 2a**)**. By IFA, the DPV gE was predominantly detectable in the juxtanuclear region where it might be the TGN, a site for viral final envelopment and sorting of virions to cell junction **(**Fig. 2c**)**^24,25^.

DPV gE played an important role in regulating the spread of the virus between adjacent cells. Cell‐to‐cell spreading not only facilitated rapid viral transmission but also promote viral immune evasion^8^. Thus, nonessential glycoprotein gE fulfills an important role in DPV infection and invasion. Plaque morphology assays in this study showed that the deletion of *gE* reduced plaque size **(**Fig. 3b**)** and it was similar to the effect of the deletion in gE of HSV and PRV^26^. To HSV as an example, gE utilized its CT domain specifically directed nascent virions sorting to cell junctions in the infected late phase^24^. The tyrosine motifs (YXXØ) of HSV gE CT domains directed the sorting of proteins to cell junction^27^. It is worth noting that DPV gE protein also contained YXXØ (YGSY and YNSL) in the CT domain, and these two motifs may be implicated in virions sorting process^23^.

The deletion of DPV *gE* reduced viral titers and it indicated that DPV gE was essential for viral growth in DEF cells **(**Fig. 4a,b**)**. Virus growth curve analysis of PRV showed that the triple-deletion of *gE, gI* and *gM* replicated on RK13 cells with significantly lower efficiency than the wild-type virus^16^. The observed growth defect can be explained by the impaired of virion morphogenesis, as demonstrated by ultrastructural analysis. Thus, to further explore the influence of gE on viral growth, ultrastructural analysis of DPV and its mutant was performed by using electron microscopy. According to the observation of ultrastructure analysis, we found that deleting DPV *gE* resulted in a large number of nucleocapsids accumulated around intracellular vesicles with very few of them budding into vesicles **(**Fig. 6c–f**)**. It indicated that DPV gE was implicated in late stages of virion morphogenesis before the final envelopment of intracytoplasmic nucleocapsids. Different results showed in HSV and PRV. In HSV, defects of virions morphogenesis observed with *gE* single mutant were relatively subtle compared to the more severe phenotype of a mutant lacking both *gD* and *gE*^15^. In PRV, the deletion of *gE* appears to proceed with a relatively normally virions assembly process, however, the deletion of *gE* and *gM* inhibited viral maturation^16^. It indicated that in HSV and PRV, nonessential glycoproteins produced a synergistic effect on virions morphogenesis and in the absence of those nonessential glycoproteins, tegument formation around capsids still occurred but the process of nucleocapsids bound onto TGN was blocked.

According to the above phenomenon we have raised a question: why did a large number of nucleocapsids accumulate around the cytoplasmic vesicles after deletion of DPV *gE* and what the role did *gE* play in it? As we all know, the final envelopment involved binding of tegument-coated capsids to viral glycoprotein-enriched regions of the TGN as enveloped virions bud into TGN membranes^19^. The CT domains of gE could allow glycoproteins enriched at TGN^24,25^. Deleting PRV *gE* cytoplasmic tail and *gM* inhibited nucleocapsids to bind onto TGN^25^. The molecular basis for final envelopment was implicated in the interactions of capsid proteins, tegument proteins and glycoproteins^28^. Tegument proteins served as a bridge to interact with capsids on one side and the glycoproteins on the other side^29^. In HSV infected cells, gE interacted with tegument proteins including UL11, UL16, UL21, and VP22 to bind capsids anchored onto the TGN membrane and promote the occurrence of final envelopment^21,30^. Thus, deleting *gE* not only damaged the bridge functions of tegument proteins but also influenced the correct location of nucleocapsids at TGN. In this paper, results showed that deleting *gE* caused numbers of capsids accumulated around TGN vesicles, which means it may exist other proteins that located capsids onto TGN. TGN-located nucleocapsids could not bud into vesicles after deleting *gE*. We hypothesized that in the absence of *gE*, the process of nucleocapsids budding into trans-Golgi vesicles was strongly compromised. At present, the molecular basis of virus budding into the TGN membrane to acquire the final envelopment was unclear at exactly. Some papers speculated that the cytoplasmic tails of glycoproteins may make contact with tegument proteins for driving the final budding process, but others believed that the stability protein networks constructed by glycoproteins and tegument proteins on the surface of TGN membrane may allow the budding process to occur^20,28,31^.

In summary, in this study, we found that the DPV gE protein played an important role in viral infection by regulating viral cell-to-cell spreading and virion final envelopment. This paper provided us a message that DPV gE may affect virion final envelopment by promoting capsids into TGN vesicles. This work may enrich and refine the final envelopment process of alphaherpesviruses and provided detailed information for the study of the molecular mechanism of DPV.

## Methods

### Ethics statement

This study was approved by the Committee of Experiment Operational Guidelines and Animal Welfare of Sichuan Agricultural University. Experiments were conducted in accordance with approved guidelines.

### Cells

DEF cells were prepared from 9-day-old cherry valley duck embryos and maintained in Modified Eagle’s Medium (MEM, Gibco) supplemented with 10% new calf serum (NBS, Gibco) and antibiotics at 37 in 5% CO_2_.

### Antibodies

The antibodies used in this study, including the rabbit anti-CHv polyclonal antibody and rabbit anti-gE polyclonal antibody, were previously prepared in our laboratory. Goat anti-rabbit immunoglobulin G (IgG) antibody conjugated with streptavidin-biotin complex (SABC) was used as the secondary antibody for plaque morphology analysis (Bosterbio). Goat anti-rabbit IgG antibody conjugated with horseradish peroxidase (HRP) was used as the secondary antibody for the Western blot assays (BioRad). Alexa-568 goat anti-rabbit IgG (H + L) was used as the secondary antibody for the IFA(Thermo Fisher).

### Construction of BAC-CHv mutant and revertant viruses

*gE* mutant virus utilized in this study was constructed by using an infectious BAC clone containing the whole DPV Chinese virulent strain (CHv), named pBAC-CHv. The pBAC-CHv was constructed by inserting the BAC mini-F sequence and EGFP selection marker into the CHv *UL23* gene and it could be stably maintained in GS1783 and reconstituted again in DEF cells^32^. Mutagenesis was based on a markerless two-step Red recombination^17^. In brief, a linear PCR product in which a *Kan* cassette contained an *I-SceI* homing endonuclease site flanked by a 40 bp upstream homologous arm and an 80 bp downstream homologous arm, which was amplified separately from the upstream and downstream of the *gE* coding sequence being deleted, was electroporated into GS1783 bacteria containing pBAC-CHv. All products were amplified by PCR using the pEPkan-S vector as the template. The forward and reverse primers used in this step are listed in Table 2. The first lambda Red recombination step promoted the replacement of the wild-type target gene sequences with the marker cassette. Subsequently, en passant recombination was performed by utilizing a duplicated sequence within the homologous arms of the inserted PCR products and an *I-SceI* homing endonuclease site to remove the complete marker cassette in a second lambda Red recombination step. The deletion of target genes was verified by sequencing with identification primers **(**Table 2**)**.

**Table 2:** Primers used in this work.

Primer name	Sequence 5′-3′	Product
∆gE-F	ATACTGCCGGCCAGACTACGGAACCTCAACAATTGGTACGTAGGGATAACAGGGTAATCGATTT	Kana gene flanked by homology arms of gE
∆gE-R	TAACTATTTCACTAGTGAGTCATTAGTTCAACATCCATGACGTACCAATTGTTGAGGTTCCGTAGTCTGGCCGGCAGTATGCCAGTGTTACAACCAAT	
RgE-F	TTGGAGTACTAAACACCAACATACTGCCGGCCAGACTACGGAACCTCAACAATTGGTACGATGATGGTTACTTTTATATC	gE gene flanked by homology arms of gE
RgE-R	TGAGTCATTAGTTCAACATCCATGATCAGATGCGGAAACTAGATT	
RgE-Kana-F	TCATGGATGTTGAACTAATGACTCACTAGTGAAATAGTTACCTGTATTACTAGGGATAACAGGGTAATCGAT	Kana gene flanked by homology arms of gE
RgE-Kana-R	CAGGTGTCGGCCTAATATACCTGTGCATTAGTAATACAGGTAACTATTTCACTAGTGAGTCATTAGTTCAACATCCATGATGTTACAACCAATTAACCA	
gE-F	TCTCAAGACGCTCTGGAATC	gE gene identification primers
gE-R	AGCGAGTACTTCTCTGCGTC	

To confirm that nonspecific mutations occurred in the rest of the mutated genomes, the *gE* revertant virus was constructed. The construction of the revertant virus was also based on the markerless two-step Red recombination. Briefly, the coding sequence of the *gE* gene was produced by PCR using the CHv genome as a template. The forward and reverse primers used in this step were listed in Table 2. The forward primer contained a 60 bp homologous arm located upstream of the *gE* gene, and the reverse primer contained 25 bp homologous located downstream of the *gE* gene. The *Kan* cassette with an *I-SceI* homing endonuclease site was amplified using the pEPkan-S vector as the template. The forward primer contained a 50 bp sequence located downstream of the *gE* gene, which contained 25 bp that were homologous to the reverse primer amplifying the coding sequences of the *gE* gene **(**Table 2**)**. The reverse primer of the *Kan* cassette contained 80 bp that were located downstream of the *gE* gene. The *gE* gene and *Kan* cassette coding sequence products were fused by PCR and then electroporated into GS1783 bacteria containing pBAC-CHv-∆gE. The subsequent steps were similar to those used to construct the mutants. The target gene was sequenced with identification primers **(**Table 2**)**. The correctly sequenced plasmid was transfected into DEF cells, producing the virus BAC-CHv-∆gE and BAC-CHv-∆gE Rev.

### Western blot

To analyze the effect of the mutation on gE expression, DEF cells were infected with BAC-CHv and the *gE* mutant and revertant viruses, and protein lysates were collected in RIPA buffer at 48 h post-infection. The lysis buffer was supplemented with PMSF. Samples were analyzed by Western blot using the rabbit anti-gE antibody and goat anti-rabbit IgG antibodies.

### Indirect immunofluorescence assay

DEF cells growing in 6-well dishes with glass coverslips were infected with BAC-CHv and the *gE* mutant and revertant viruses at an MOI of 0.01. After 48 h, the cells were washed and then fixed with 4% paraformaldehyde overnight at 4 °C. The cells were then washed in phosphate buffered saline (PBS) containing 0.01% Tween 20 (PBS-T), permeabilized with 0.25% Triton X-100 in PBS for 30 min, and washed three times with PBS-T before being incubated with 5% BSA (biotechnology) overnight at 4 °C. The cells were then incubated with the rabbit anti-gE polyclonal antibody overnight at 4 °C, washed three times with PBS-T, and incubated with Alexa-568 goat anti-rabbit IgG (Thermo Fisher) for 1 h at 37 °C. The cells were then washed with PBS-T for five times, incubated with DAPI for 15 min at 37 °C and washed again with PBS-T. The cells were photographed using a Nikon ECLIPSE 80i inverted fluorescent confocal microscope with a SpotFlex image restoration system. Images were processed using Photoshop software.

### Viral growth curve

Analysis of the viral growth curve was performed as follows. Briefly, DEF monolayer cells grown in 24-well cell culture dishes were infected with BAC-CHv and the *gE* mutant and revertant viruses at an MOI of 0.01. The viruses were allowed to penetrate for 2 h at 37 °C in 5% CO_2_. Next, the culture medium was removed, and the cells were washed with PBS (pH 7.4) and then overlaid with MEM containing 2% NBS. At 24, 48 and 72 h, the supernatants and cell pellets were separately harvested and collected. The samples were frozen and thawed three times, and viral titers were determined by the TCID_50_ on DEF cells. All experiments were repeated three times.

### Plaque morphology

Visual analysis of plaque morphology was performed as follows. Briefly, near-confluent (85 to 90%) DEF monolayer cells in 6-well plates with glass coverslips were infected with BAC-CHv and the *gE* mutant and revertant viruses at an MOI of 0.01. The viruses were allowed to penetrate for 2 h at 37 °C in 5% CO_2_. Thereafter, the culture medium was removed, and the cells were washed three times with PBS (pH 7.4). The DEF cells were incubated for 24 h in MEM containing 1% methylcellulose and 2% NBS. The culture medium was removed again, and the cells were washed three times with PBS. The cells were then fixed with 4% paraformaldehyde overnight at 4 °C, washed three times with PBS-T and permeabilized with 30% H_2_O_2_ and methanol at a ratio of 1 to 50 for 30 min. The cells were incubated with 5% BSA (Biotechnology) for 30 min and then incubated with the rabbit anti-CHv polyclonal antibody and goat anti-rabbit IgG antibody conjugated to SABC. After washing three times with PBS-T, the cells were colorized by the addition of diaminobenzidine (DAB, BosterBio). Photographs of viral plaques were taken at 40× magnification, and 100 randomly selected plaques were imaged for the viruses under consideration.

### Electron microscopy

All samples were prepared for transmission electron microscopy examination according to previous reports^33^. DEF cells were infected with BAC-CHv and its mutant and revertant viruses at an MOI of 5. After 20 h, the DEF cells were washed with PBS, scraped and centrifuged at 3000 rpm for 15 min. The cells were fixed in 2.5% glutaraldehyde, washed with PBS, fixed in 1.0% osmium tetroxide and then subjected to stepwise dehydration in acetone. The samples were embedded in epoxy resin 618 and polymerized at 80 °C for 72 h. Then, 50 nm ultrathin sections were prepared, collected on grids, stained with uranyl acetate and lead citrate, and examined with the Tecnai G^2^F20 transmission electron microscope.

## Supplementary information

Supplementary information.
