# Supplementary material for: Duck plague virus gE serves essential functions during the virion final envelopment through influence capsids budding into the cytoplasmic vesicles
Source: Sci Rep. 2020 Mar 27;10:5658. doi: 10.1038/s41598-020-62604-9 (PMC7101321; doi:10.1038/s41598-020-62604-9)
Supplement: Supplementary file 1 — Supplementary information. [file 41598_2020_62604_MOESM1_ESM.pdf]

Duck plague virus gE serves essential functions during the virion final envelopment through influence capsids budding into the cytoplasmic vesicles

Tian Liu[1,2,3]†, Mingshu Wang[1,2,3]†, Anchun Cheng[1,2,3]*, Renyong Jia[1,2,3], Qiao Yang[1,2,3], Ying Wu[1,2,3], Mafeng Liu[1,2,3], Xinxin Zhao[1,2,3], Shun Chen[1,2,3], Shaqiu Zhang[1,2,3], Dekang Zhu[2,3], Bin Tian[1,3], Mujeeb Ur Rehman[1,3] , Yunya Liu[1,2,3], Yanling Yu[1,2,3], Ling Zhang[1,2,3], Leichang Pan[1,3], and Xiaoyue Chen[2,3]

[1]Institute of Preventive Veterinary Medicine, Sichuan Agricultural University, Wenjiang 611130, People's Republic of China.

[2]Avian Disease Research Center, College of Veterinary Medicine of Sichuan Agricultural University, Wenjiang 611130, People's Republic of China.

[3]Key Laboratory of Animal Disease and Human Health of Sichuan Province, Sichuan Agricultural University, Wenjiang 611130, People's Republic of China.

* Correspondence: chenganchun@vip.163.com

† These authors contributed equally to this work

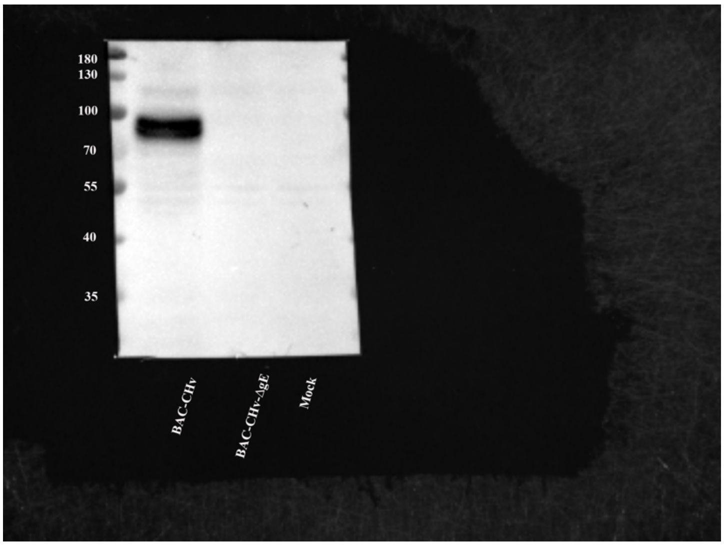

Fig 2a: Western blot analysis of gE protein expression of BAC-CHv and BAC-CHv-ΔgE.

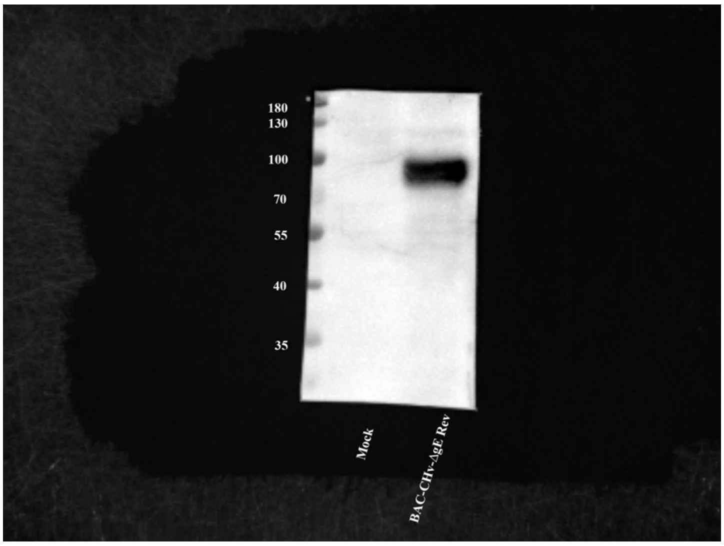

Fig 2b: Western blot analysis of gE protein expression of BAC-CHv-ΔgE Rev.
